# Case Report: Using ultrasound to prevent a broken catheter from migrating to the heart.

**DOI:** 10.12688/f1000research.11206.1

**Published:** 2017-05-03

**Authors:** Pieter J. Schraverus, Suzanne van Rijswijk, Pieter Roel Tuinman

**Affiliations:** 1 Department of Intensive Care, Vrije Universiteit Medical Centre, Amsterdam, Netherlands

**Keywords:** broken catheter, ultrasound, echography

## Abstract

Peripheral intravenous (IV) catheters can break off while still in the patient, with possible detrimental effects such as upstream migration to the heart. These catheters have probably been damaged by the needle during a difficult insertion. A peripheral IV catheter was removed in a 90 year old patient and only half of the catheter was retrieved. By using ultrasound examination the remaining part of the IV catheter was identified, and retrieved surgically, before it could migrate towards the heart. This case report suggests that ultrasound should not only be used for difficult placement of a peripheral IV catheter, but can also be used when removal is complicated.

## Introduction

Peripheral intravenous (IV) catheters are given every day to many patients, without much attention given to possible complications. The complications are usually minor, for instance phlebitis or subcutaneous injection of solutions. However, when placement is difficult and the needle of the IV catheter is reinserted for another attempt, the needle can cut the catheter and damage it in such a way that it might break while inside the vein. These fragments can migrate to the right side of the heart, both atrium and ventricle, evidenced by reports in the literature ^1, 2^.

## Case report

We present the case of a 90 year old caucasian patient where only half of the peripheral IV catheter was retrieved after removal. By using ultrasound the remaining part of the catheter was identified and removed.

**Figure 1.**
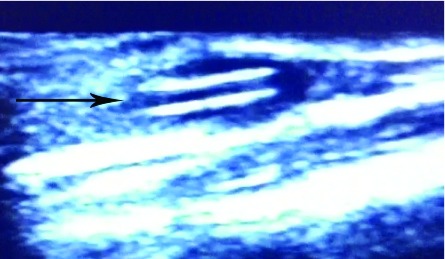
Ultrasound image of the lower arm. The black arrow indicates the broken catheter.

**Figure 2.**
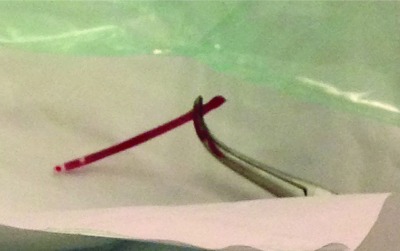
Broken distal part of the peripheral catheter after retrieval.

The patient was admitted to the hospital with a contained ruptured aneurysm of the abdominal aorta. The patient underwent emergency surgery and an aortic bifurcation prosthesis was placed. According to the anaesthesiologist who cared for the patient, the peripheral catheter was used to administer the anaesthetics and induction of anaesthesia went as planned. After induction, a central venous catheter was placed and the peripheral catheter was no longer used. Post operatively the patient was admitted to the intensive care unit (ICU) where the peripheral catheter was removed and only the proximal half of the catheter came out. We examined the arm but could not palpate the remaining part of the catheter. Ultrasound examination, performed by the ICU resident, showed an echogenic hollow tube ( Figure 1), eight centimeters proximal of the insertion site of the catheter. The surgeon made a small incision and the remaining part of the catheter was removed ( Figure 2). We were not able to trace the person who placed the IV catheter, to evaluate the technique used.

## Discussion

This case describes the succesful use of ultrasound as a diagnostic tool for difficulties encountered after peripheral IV catheter removal.

We hypothesize that the needle was reinserted in the catheter during placement because placement was difficult. By doing so, the needle may have cut the distal part of the catheter. Reinserting a needle back into a catheter can be dangerous, and there is a risk of damaging the catheter or cutting it off and thereby allowing the free part of the catheter to migrate to the heart, a consequence that could be disastrous and would make removal of the catheter far more difficult ^3, 4^. The use of ultrasound made a quick diagnosis and enhanced treatment possible, thereby preventing further complications.

## Consent

Written informed consent for publication of clinical details and clinical images was obtained from the patient.
