# Supplementary material for: Case Report: Using ultrasound to prevent a broken catheter from migrating to the heart
Source: F1000Res. 2017 May 3;6:616. [Version 1] doi: 10.12688/f1000research.11206.1 (PMC5500861; doi:10.12688/f1000research.11206.1)
Supplement: Peer review history

# Peer review: Referee response for version 1

**DOI:** 10.5256/f1000research.12090.r22448

**Published:** 2017-07-06

**Authors:** Joris Lemson

**Affiliations:** 1 Department of Intensive Care, Radboud University Medical Centre, Nijmegen, Netherlands

This case report shows the usefulness of ultrasound when a broken intravenous canula had migrated away from the insertion location. The case is adequately described and illustrated and the message is clear. It would be of interest to know if ultrasound devices that are often used at intensive care units are also adequately equipped for searching foreign bodies.

I have read this submission. I believe that I have an appropriate level of expertise to confirm that it is of an acceptable scientific standard.

# Peer review: Referee response for version 1

**DOI:** 10.5256/f1000research.12090.r22447

**Published:** 2017-05-08

**Authors:** Michiel Justinus Blans

**Affiliations:** 1 Department of Internal Medicine and Intensive Care, Rijnstate Hospital, Arnhem, Netherlands

This case report shows that ultrasound can also be used for the described indication. It is well structured and the photo material is clearly showing the described content. Being a short case report no literature review is needed. It is important that modern doctors learn to use all possible aspects of point-of-care ultrasound (as briefly stated, also for the insertion of iv catheters).

I have read this submission. I believe that I have an appropriate level of expertise to confirm that it is of an acceptable scientific standard.
